# C-reactive Protein: An Inflammatory Biomarker and a Predictor of Neurodegenerative Disease in Patients With Inflammatory Bowel Disease?

**DOI:** 10.7759/cureus.59009

**Published:** 2024-04-25

**Authors:** Simona Muresan, Mark Slevin

**Affiliations:** 1 Internal Medicine IV, George Emil Palade University of Medicine, Pharmacy, Sciences and Technology, Targu Mures, ROU; 2 Center for Advanced Medical and Pharmaceutical Research (CCAMF), George Emil Palade University of Medicine, Pharmacy, Sciences and Technology, Targu Mures, ROU

**Keywords:** crp, c-reactive protein, neurodegenerative diseases, neuroinflammation, chron’s disease, ulcerative colitis (uc), monomeric c-reactive protein

## Abstract

Inflammatory bowel disease (IBD) refers to two chronic conditions of the digestive tract: ulcerative colitis (UC) and Crohn’s disease (CD), representing a progressive inflammatory process that mainly occurs in the gut, with frequent extra-intestinal manifestations. Even if remission is periodically obtained for some patients, the histological activity and digestive symptoms may continue, maintaining a persistent systemic inflammation that could induce further extra-intestinal complications and contribute to the development of neurodegenerative disease.

C-reactive protein (CRP) is an acute-phase reactant that is widely accepted as a dominant serum biomarker in IBD. CRP consequently activates the complement cascade, supports the release of pro-inflammatory cytokines, and the clearance of microbial pathogens. All these processes facilitate further processes, including atherosclerosis and hypercoagulability, alteration of the intestinal microbiota, and the increased permeability of the intestinal barrier for neurotoxic substances produced by gut microorganisms, due to the presence of a high level of lipopolysaccharides. For IBD, the connection between intestinal inflammation and central nervous system inflammation could be explained through the activity of the vagus nerve, a carrier of cytokines, CRP, and toxic materials to the brain, potentially inducing vascular lesions and damage of the glial vascular unit, with further risk for degeneration within the central nervous system.

CRP is a key marker for IBD pathogenesis and is able to dissociate into its monomeric form, mCRP, on contact with activated cell and tissue components via the systemic circulation. We hypothesize that the chronic inflammatory process within IBD could initiate neuroinflammation and neurodegeneration, and therefore, further investigation of the significance of chronically raised plasma of CRP and mCRP in patients with IBD is warranted, as it may represent a critical predictive factor associated with a later neurodegenerative risk. Any future initiative aimed at pharmacologic modulation of CRP (e.g., blocking CRP-mCRP dissociation), could represent a new therapeutic approach protecting against intestinal inflammation and concomitantly reducing the risk of neuroinflammation, neurodegeneration, and cognitive decline.

## Introduction and background

Classical features of IBD

Inflammatory bowel disease (IBD) refers to two chronic conditions of the digestive tract: ulcerative colitis (UC) and Crohn’s disease (CD), representing a chronic and progressive inflammatory process that mainly occurs in the gut, with frequent extra-intestinal manifestations [1]. Although the etiopathogenesis remains unclear, the immune response to the saprophytic gut flora and external factors acting on patients with a specific genetic profile are considered the most likely triggers [2]. The young age of onset and the oscillatory evolution with frequent flares significantly impact the quality of life of affected individuals and require comprehensive dynamic management from diagnosis to monitoring and treatment to minimize bowel damage, hospitalization, surgeries, and ultimate disability [1,3].

Because mucosal healing is imposed as the treatment goal for patients with IBD, endoscopy with biopsy has become the gold standard for diagnosis and IBD assessment in patients [1]. However, due to its limitations in terms of accessibility, cost, risk or complications, and patient compliance, more emphasis has been placed on biomarkers over the last decade [4].

Biomarkers in IBD: standard tests and new biological markers of inflammation

IBD biomarkers represent quantifiable characteristics of biological processes that should correlate with clinically meaningful endpoints of the disease, such as symptoms, progression, activity, resolution, and dynamic changes related to treatment response; as well as endoscopy and imaging reference points. These markers can therefore be effectively applied as important reference points within daily practice [4]. In recent years, various potential IBD biomarkers have emerged, detectable in blood serum, fecal markers, or in some cases, both.

Due to the acute phase response in IBD patients, significantly increased concentrations of proteins involved in coagulation and fibrinolysis (such as fibrinogen and plasminogen), components of the complement system, proteinase inhibitors (α1-antitrypsin, α1-antichymotrypsin), transport proteins (haptoglobin, ceruloplasmin), and C-reactive protein (CRP) or ferritin are found in both UC and CD [5]. In addition, various pro-inflammatory cytokines that could serve as biomarkers and/or indicate the effectiveness of therapy are elevated, particularly tumor necrosis factor-α (TNF-α), interferon-β (IFNβ), transforming growth factor-β (TGFβ), and interleukins (IL-1β, IL-6, IL-8, IL-12, IL-17, and IL-23) [6]. The innate hyperactive chronic immune response in IBD is the primary exacerbator of tissue injury and the main cause of symptoms in patients, and recently, new therapies specifically designed to block the effects of TNF-α, such as infliximab, have shown promise by reducing tissue-associated inflammation in selected individuals [7].

The extent of inflammation can also be measured indirectly via increased erythrocyte sedimentation rate (ESR), often partnered with serum CRP. Most recently, leucine-rich alpha-2 glycoprotein (LRG) has emerged as a new and potentially more sensitive serum diagnostic biomarker that correlates with disease activity in IBD and remission in CD [8]. However, to date, there are still only a few blood biomarkers of inflammation fully characterized and validated in IBD, with CRP and ESR being the most widely available and used [5].

There remains a need to identify new markers or panels that can accurately predict disease progression, sensitivity to treatment, and further understanding of the pathobiological mechanisms of onset and development that could result in the formulation of novel therapeutic interventions. Here, we describe the potential importance of CRP as a direct moderator in IBD and hypothesize for the first time a further role in predicting IBD-associated risk of development of AD.

## Review

IBD and dementia

Neurodegenerative Disease: A Serious Consequence in the Evolution of IBD Patients

IBD represents a digestive tract pathology with an incidence that is increasing at a rapid rate. With its continual growth on a global scale, IBD has become an immense economic strain on health systems in recent years [9]. Beyond the new paradigm for managing these clinical cases, which includes "risk stratification" of patients, the application of highly effective therapy earlier in the disease course for high-risk patients, and the reassessment of response using objective outcome measures at specified points in time, a focus has been placed on studying the association of IBD with comorbidities such as cardiovascular and neurological diseases, to optimize the evolution of these patients where possible [5].

Dementia is a progressive neurodegenerative disease that leads to a decline in cognitive ability, caused by various neurological disorders. It has a global estimated prevalence of approximately 7% among individuals aged 65 or older [10,11]. The primary cause of dementia is neurodegenerative disorders, such as Alzheimer's disease (AD), dementia with Lewy bodies, and Parkinson’s disease [12]. Recent years have seen contradictory studies published regarding the association between IBD and dementia (more specifically AD), offering different explanations for the pathophysiological processes involved.

Within the Atherosclerosis Risk in Communities (ARIC) cohort study, inflammatory biomarkers including CRP were measured during mid-adulthood. The study demonstrated that systemic inflammation is associated with increased cognitive decline among patients followed for up to 20 years [13]. Zhang et al. (2021) showed that IBD patients were diagnosed with dementia at an earlier age compared to matched controls (mean age 76 vs 83) and the risk was positively associated with the chronicity of the IBD diagnosis [14,15]. A study by Zuin et al. (2022), which included more than 14,000 patients with IBD and a follow-up period of over 20 years, showed that subjects with CD had an increased risk for dementia, whereas patients with UC did not show statistical significance for this association. Particularly, males with CD presented a higher risk for dementia compared with females [16]. A meta-analysis conducted by Cooper et al. (2023), based on eight different studies, indicated that dementia and cognitive impairment are 1.91 times more prevalent in patients with IBD [17].

IBD therapy, including immunosuppressants and TNF blockers, plays a major role in controlling the severity of inflammation and indirectly has a role in the development of neurodegenerative diseases. The evaluation of exposure to therapy, included in the updated meta-analysis, suggests a possibly lower risk of AD in IBD patients with a history of effective medication treatment, compared to those without exposure to medication [18]. A new perspective was established following recent genome-wide association studies (GWAS) that found no significant genetic correlation of IBD with AD or cognitive traits; conclusions indicated that some ‘risk’ genes for AD could be protective against IBD and vice versa [19].

The Pathogenic Mechanism of Neurodegeneration in IBD

IBD is a chronic inflammatory disease that affects the intestinal and parenteral areas. Even if remission is periodically obtained for some patients, the histological activity and extra-digestive symptoms may continue, maintaining a persistent systemic inflammation that could induce extra-intestinal complications and perhaps contribute to the development of neurodegenerative diseases [11].

The pathogenic mechanism that could explain the increased risk of dementia in patients with IBD is multifactorial. The major contributing factor is chronic inflammation, but other elements including accelerated atherosclerosis and hypercoagulopathy should not be ignored [12]. In IBD, CRP, adhesion molecules, and inflammatory cytokines are overexpressed both locally and systemically. These chronic inflammatory factors contribute to vascular endothelial dysfunction, impaired fibrinolysis, activation of the coagulation cascade, and abnormal platelet function resulting in increased arterial and venous thrombosis [9]. Consequently, IBD is associated with a higher risk of thromboembolic events and stroke due to thrombocytosis, coagulation abnormalities, hyperlipidemia, and hyperhomocysteinemia, thus contributing to the development of vascular dementia, which represents at least 20% of all dementia cases [12].

Alterations in intestinal microbiota due to local inflammation might be a trigger stimulating excessive inflammation and partially explaining the link between IBD and dementia [15]. Studies on postmortem brain samples from AD patients have identified the presence of bacterial lipopolysaccharides (LPS) generated by gut microbes. This evidence suggests that the gut microbiota is involved in regulating microglial activation, neuroinflammation, and potential subsequent dysregulation of the glial and neurovascular units in AD. Both IBD and AD patients were also found to have decreased microbiome diversity [20]. This common peculiarity explains the reduced chance for these patients to produce beneficial anti-inflammatory metabolites such as short-chain fatty acids (SCFAs), certain bile acids (tauroursodeoxycholic acid), and ligands for aryl hydrocarbon receptors. These molecules are usually capable of crossing the blood-brain barrier (BBB) and modulating the inflammation in the central nervous system [15]. Without them in adequate quantities, neuroinflammation may be unavoidable.

A further relevant pathogenic mechanism described in IBD is intestinal leakage, caused by alteration of the intestinal barrier due to local inflammation. This allows toxic substances (and even inflammatory factors) produced by intestinal microorganisms to enter the more permeable circulatory system, potentially traversing the blood-brain barrier (BBB) with a concomitant predisposition towards a neuroinflammatory phenotype within the central nervous system (CNS) [15].

Involvement of the Vagus Nerve and the Gut-Brain Axis

The connection between intestinal inflammation and neuronal inflammation could be considered the vagus nerve, which provides a link between the enteric nervous system (ENS) and the central nervous system (CNS). In support of this hypothesis, Walker et al. (2019) described the relationship between systemic inflammation, activity of the vagus nerve, and the reactive status of brain endothelium and glia associated with defective neurovascular unit (NVU) function and the build-up of toxic β-amyloid and tau [21]. Stimulation of the vagus nerve reduced systemic inflammation, circulating CRP, and symptoms in a series of Crohn’s patients [22], while electroacupuncture-mediated activation of the vagal-adrenal axis in a murine model of transgenic Alzheimer's disease significantly reduced neuroinflammation via NOD-, LRR-, and pyrin domain-containing protein 3 (NLRP3) and IL-1β perturbation, concomitantly protecting against cognitive decline [23].

Direct evidence of the involvement of the vagus nerve in the transmission of toxic material to the brain was shown by Sun et al. (2020) [24]. They administered β-amyloid (1-42 oligomers) into the gastric wall of mice and demonstrated migration of the β-amyloid into cholinergic neurons within hours, later followed by transfer to the proximal colon within one month. Most surprisingly, deposits were identified both in the vagus nerve and in the brain within 12 months of the initial treatment, concomitant with the appearance of AD-like symptoms represented by changes in cognitive ability and behavior.

**Figure 1:**
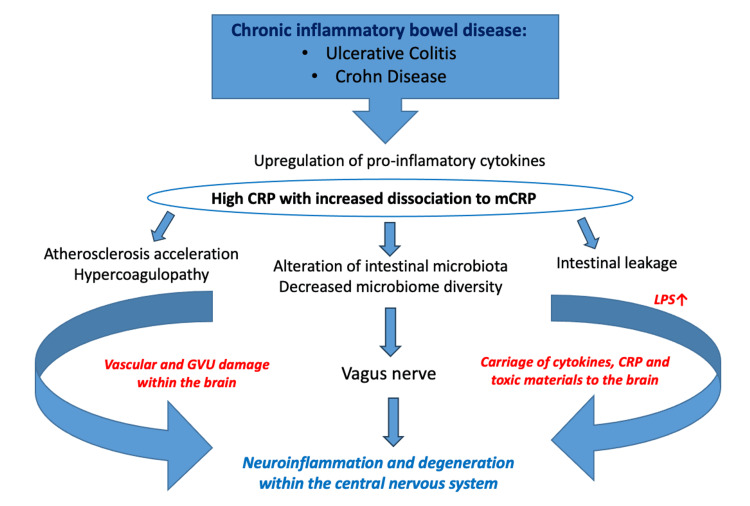
Mechanism leading to neuroinflammation and neurodegeneration in chronic inflammatory bowel disease Ulcerative colitis and Crohn’s disease are characterized by chronic inflammation of the intestinal tissue and parenteral areas, with overexpression of inflammatory cytokines, high levels of CRP, and possibly increased subsequent dissociation to mCRP. The chronic inflammation supports the activation of processes such as atherosclerosis and hypercoagulability, alteration of the intestinal microbiota, and an increased permeability of the intestinal barrier to neurotoxic substances produced by gut microorganisms, facilitated by the presence of high levels of lipopolysaccharides. The link between the inflammation at the level of the enteric nervous system and the central nervous system is represented by the vagus nerve. It acts as a ”carrier” of cytokines, CRP, and toxic materials to the brain, potentially inducing vascular lesions and damage to the glial vascular unit, with further risk for degeneration and dementia. Created with Microsoft PowerPoint. CRP: C-reactive protein; mCRP: monomeric C-reactive protein; GVU: glial vascular unit; LPS: lipopolysaccharides

Therefore, chronic IBD may participate in and/or be a causative factor in the development of neurodegenerative diseases, whereby localized inflammation of the bowel leads to systemic production of inflammatory cytokines that can penetrate the CNS, directly as a result of increased permeability and damage to the BBB, or through transmission via the vagus nerve (Figure 1). Both β-amyloid and α-synuclein have been shown to be transferred via the gut wall to the vagus nerve. Given the potential significance of CRP in IBD and amyloid plaque development within the brain, we examine the potential role of CRP in the mediation of these processes [25-27].

CRP: a potential prognostic marker for IBD due to its role in disease pathogenesis

CRP: Background and Synthesis

CRP is an acute-phase reactant that is widely accepted as a dominant serum biomarker in IBD trials [2,5]. It is well known that during all stages of inflammation, IL-1β and tumor necrosis factor (TNF-α) activate macrophages and T cells to secrete interleukin-6 (IL-6). IL-6 is the main stimulator of hepatocyte-mediated production of CRP. CRP consequently activates the complement cascade, supports the further release of pro-inflammatory cytokines, and facilitates the clearance of microbial pathogens [5]. Other sites for CRP production have been described, including peripheral lymphocytes, neurons of patients with AD, and in the thickened intima of atherosclerotic plaques [28]. CRP has a short half-life of approximately 19 hours, which is independent of any physiological or pathophysiological circumstances or of the concentration of CRP in the serum. This property ensures that the concentrations quickly decrease once the acute-phase stimulus disappears, making CRP a very valuable marker to detect and follow-up on inflammation [29].

CRP as a Biomarker for Prognostic and Diagnostic Stratification in IBD

CRP was studied for both IBD subtypes (UC and CD) in four principal ways: as a potential tool for differential diagnosis between UC and CD, as a marker for endoscopic or histological disease activity, as a predictor for recurrences, and as a marker of risk for surgery and for treatment response [1]. Studies revealed that CRP is an efficient monitoring marker to assess patients in the active phase of IBD and is appropriate for evaluating the efficiency of treatment through repeated measurement. However, due to its more limited accuracy in patients with lower activity, the poor prediction of mucosal healing by using CRP monitoring alone indicates a preference for the use of multiple biomarkers [30].

CD patients generally have higher CRP production than patients with UC, along with a higher concentration of IL-6 [2,31]. Some possible explanatory mechanisms involve the accumulation of mesenteric fat, a major site of IL-6 and TNF-α synthesis in CD patients, as well as the association between disease flare-ups and significant bacterial migration during the extensive transmural inflammation in CD compared with mild to moderate mucosal inflammation in UC [2,31]. Regarding the risk of surgery, an increased level of high-sensitivity CRP (hsCRP) at diagnosis might be used as a predictive factor in evaluating the risk of surgery in CD [32,33].

The protocols used in hospitalized patients for monitoring severe disease activity (CD or UC) suggest that clinical features should be evaluated daily, and CRP should be measured every one to two days due to its correlation with the response to therapy [4,34,35]. Due to the close relationship between TNF-α, IL-6, and CRP concentrations, and because mucosal inflammation lags behind the normalization of biomarkers, CRP could be used to assess pharmacodynamics. In patients with severe colitis treated with infliximab, the neutralization of TNF-α induced by the drug should result in a fast decline in CRP concentration due to downstream effects on IL-6. The absence of this response could be recorded as rapid drug clearance and low drug concentrations, sustaining the concept of adaptive doses, where infliximab could be dosed based on CRP serum level [5,36].

The literature, therefore, strongly supports the role of CRP as a biochemical and therapeutic marker; its pathobiological significance for chronic and progressive intestinal inflammation remains an important pillar for future approaches. Research focused on examining the association between CRP expression in patients with IBD and their comorbidities could provide critical insight into the development of novel therapeutic strategies.

The Link Between IBD, Monomeric CRP (mCRP), and Dementia

mCRP consists of the insoluble, dissociated monomers of the native molecule and is produced in situ when CRP comes into contact with activated cell and tissue components via the systemic circulation. The breakdown of the disulfide bonds holding the monomers together occurs in contact with lysophosphocholine-phospholipase C of the cell membrane (including fragments such as platelets and microparticles), and the reaction is irreversible [37]. Over the last few years, the potent pro-inflammatory biological activity of mCRP has been established, with a critical role demonstrated in the pathological initiation and progression of inflammatory diseases with vascular dysfunction, including atherosclerosis [38], neurodegenerative conditions such as AD [39], and stroke [40]. In particular, mCRP has been identified in association with damaged brain tissue parenchyma in infarcted regions after stroke, and as a key component in developing β-amyloid plaques [41]. Both animal models of AD and in vitro evidence confirm pathobiological mechanisms through which mCRP contributes to neurovascular dysfunction, including prolonged activation of inflammation and neuroinflammation, NVU and BBB dysfunction, increase in vascular permeability, as well as stimulation of tau hyper-phosphorylation, fibril formation, and plaque generation [42,43].

While the expression of mCRP within chronic IBD has not been ascertained so far, indirect evidence suggests that this protein may be a pro-inflammatory driver in conditions such as IBD, increasing susceptibility to the development of AD. In certain autoimmune diseases such as CD, it is known that in inflammatory states, there is an oxidative stress-mediated over-production of cellular microparticles/vesicles, and these have been shown to allow mCRP to 'piggyback' upon them, supporting effective circulation and deposition at distant vascular permeable sites [44,45]. Therefore, this could explain the potential association between elevated CRP levels during chronic inflammatory conditions and an increased CRP dissociation to mCRP, with a concomitant increase in BBB permeability. In chronic inflammatory conditions, as seen in autoimmune diseases, there could be a constant and subsequent infiltration of mCRP into the brain, where its capability to disrupt the glial vascular unit and induce neuroinflammation could act as potential triggers for AD, as well as a mediator of more rapid progression of AD and dementia, together with neurocognitive decline in general [46].

## Conclusions

CRP, and especially mCRP, play a significant role in the inflammatory processes localized at the intestinal and central nervous system levels. Further research should investigate the significance of chronically raised plasma mCRP in patients with IBD, to establish if this represents a critical factor associated with a later neurodegenerative risk. Considering that no relationship between native CRP systemic concentration and mCRP concentration was demonstrated in recent studies, a reliable quantitative method is also required that will identify the blood concentrations of this protein. Any further initiative to pharmacologically modulate the involvement of this protein in the pathogenic pathway of IBD (for example, by blocking CRP-mCRP dissociation or mCRP cell membrane adhesion), could represent a new therapeutic approach able to slow down or inhibit the evolution of intestinal inflammation and to prevent neuroinflammation and the associated risk of dementia.
